# Genome-Wide DNA Methylation Analysis and Functional Validation of Litter Size Traits in Jining Grey Goats

**DOI:** 10.3390/genes15030353

**Published:** 2024-03-12

**Authors:** Cunming Yang, Junmin He, Jingyi Mao, Yifan Ren, Guifen Liu, Chen Wei, Guoping Zhang, Kechuan Tian, Xixia Huang

**Affiliations:** 1 College of Animal Science, Xinjiang Agricultural University, Urumqi 830052, China; yangcunming0405@163.com (C.Y.); 15136225372@163.com (Y.R.); 2 Institute of Animal Science and Veterinary Medicine, Shandong Academy of Agricultural Sciences, Jinan 250100, China; hejunmin330@163.com (J.H.); m2797611850@163.com (J.M.); liuguifen126@126.com (G.L.); weichenchen1989@126.com (C.W.); gpzhangsaas@163.com (G.Z.)

**Keywords:** DNA methylation, gene expression, goat ovary, prolificacy

## Abstract

DNA methylation (DNAm) is associated with the reproductive system. However, the genetic mechanism through which DNAm regulates gene expression and thus affects litter size in goats is unclear. Therefore, in the present work, genome-wide DNAm profiles of HP and LP Jining Grey goat ovary tissues were comprehensively analyzed via WGBS, and RNA-Seq data were combined to identify candidate genes associated with litter size traits in the Jining Grey goat. Finally, BSP and RT-qPCR were used to verify the sequencing results of the key genes. Notably, the DNMT genes were downregulated at the expression level in the HP group. Both groups exhibited comparable levels of methylation. A total of 976 differentially methylated regions (DMRs) (973 DMRs for CG and 3 DMRs for CHG) and 310 differentially methylated genes (DMGs) were identified in this study. Through integration of WGBS and RNA-Seq data, we identified 59 differentially methylated and differentially expressed genes (DEGs) and ultimately screened 8 key DMGs (9 DMRS) associated with litter size traits in Jining Grey goats (*SERPINB2*: chr24_62258801_62259000, NDRG4: chr18_27599201_27599400, *CFAP43*: chr26_27046601_27046800, *LRP1B*. chr2_79720201_79720400, *EPHA6*: chr1_40088601_40088800, *TTC29*: chr17_59385801_59386000, *PDE11A*: chr2_117418601_117418800 and *PGF*: chr10_ 16913801_16914000 and chr10_16916401_16916600). In summary, our research comprehensively analyzed the genome-wide DNAm profiles of HP and LP Jining Grey goat ovary tissues. The data findings suggest that DNAm in goat ovaries may play an important role in determining litter size.

## 1. Introduction

The Jining Grey goat, which originated in Shandong Province, China, is known for its high fecundity. Additionally, the Jining Grey goat represents the most famous local goat breed in the world and is an ideal animal model for studying the reproductive performance of goats [1]. Reproductive performance directly affects the economic benefits of goat farming, and the ovary, as an organ for oocyte production and reproductive hormone secretion, is directly related to litter size traits [2,3]. Existing studies have identified several genes associated with litter size traits in goats, such as *DNAH1* [4], *CFAP43* [5], *CTSS* [6], *EPHA6* [7], and *POU1F1* [8]. However, the key molecular regulatory mechanisms remain unclear.DNA methylation (DNAm) is a type of epigenetic modification that modulates gene expression. With increasing research on epigenetic modifications, it has been shown that DNAm is associated with the reproductive system. Genome-wide DNAm profiles used to study ovarian tissues from Jining Grey goats [9], Hu sheep [10], and pigs [11] with different litter sizes. In addition, Yao X et al. [2] performed DNAm and gene expression analyses via WGBS and RNA-seq techniques and reported that DNAm affects reproduction by influencing the expression of *ITGB2* and *LAPTM4B* in Hu sheep’s ovaries. Stefano Frattini et al. [12] performed DNAm and gene expression analyses via methyl-CpG binding domain protein sequencing (MBD-seq) and RNA-seq combined with analysis and showed that specific differentially methylated genes in the ovary and hypothalamus of Saanen goats correlated with gene expression levels. However, studies on genome-wide DNAm and alterations between transcriptomes have not been conducted for HP or LP Jining Grey goats. Therefore, in the present study, WGBS and RNA-seq were combined to investigate the epigenetic regulatory mechanisms of DNAm in the ovaries of HPs and LP Jining Grey goats.

This research aimed to reveal the epigenetic regulation of DNAm at different levels of prolificacy in Jining Grey goats. We used WGBS to study the DNAm profiles of ovarian DNA from HPs and LP Jining Grey goats during the estrous period. Combining these findings with previous RNA-seq data, we explored the effects of DNAm on gene expression through joint analysis of DNAm and gene expression and screened key candidate genes that may be related to litter size traits in goats. In addition, our research provides a reference for exploring the potential regulatory mechanism of litter size traits in Jining Grey goats at the DNAm level.

## 2. Materials and Methods

### 2.1. Animals and Sample Collection

The experimental protocols and animal ethics were approved by the Ethics Committee of the Institute of Animal Science and Veterinary Medicine, Shandong Academy of Agricultural Sciences (IASVM-2022-003). The experimental animals were obtained from Jining Grey goats from the National Jining Grey Goat Breeding Farm (Jiaxiang, Jining, Shandong, China). Similarly, eight ewes (3–4 years of age) with the same feeding conditions who were healthy and disease-free were randomly selected and classified as exhibiting high prolificacy (HP, litter size ≥ 3 in three consecutive litters, *n* = 4) or low prolificacy (LP, litter size = 1 in three consecutive litters, *n* = 4). First, we used a vaginally embedded CIDR pessary to treat experimental animals for simultaneous estrus. The date of CIDR pessary implantation was recorded as Day 0 and 14 days after implantation in the vagina. The pessary was withdrawn, and 300 IU pregnant mare serum gonadotropin (PMSG) was injected intramuscularly into the neck to determine the estrus status of ewes using the method used to test the estrus of ewes and vaginal examination. The ewes were slaughtered within 12 h of estrus, and the samples of the ipsilateral ovary were collected immediately and kept at −80 °C for further experiments.

### 2.2. DNA and RNA Extractions 

Total RNA was extracted from ovarian tissues using TRIzol reagent (Invitrogen, Carlsbad, CA, USA), agarose gel electrophoresis was employed to determine RNA integrity, and the RNA’s purity was assessed using a NanoDrop^TM^ One spectrophotometer (Thermo Scientific, Waltham, MA, USA). The sample quality was satisfactory for the next step of the experiment. DNA samples were extracted from ovarian tissues using a TIANamp Genomic DNA Kit (Cat.#DP304; TianGen, Beijing, China). The purity of the RNA was determined using a NanoDrop^TM^ One spectrophotometer. Finally, DNA quality was determined by 2% gel electrophoresis, and the DNA was kept at −80 °C.

### 2.3. Library Preparation

Qualified genomic DNA was sheared into 200–300 bp fragments using an ultrasonic method. After DNA end repair, adenylation of the 3′ end and ligation of sequencing junctions were performed. The fragments were bisulfite-treated using an EZ ZYMO DNAm-Gold Kit, further desalted and recovered by gum cutting. Then, library fragment size selection was performed, followed by PCR amplification, to construct a sequencing library.

### 2.4. WGBS and Identification of DMRs

Qualified libraries were subjected to whole-genome bisulfite sequencing (WGBS) using the MGISEQ2000 platform (MGI Tech Co., Ltd., Shenzhen, China), and the raw reads were filtered through SOAPnuke V1.5.6. Reads containing splice contamination were first removed, after which reads with an N content greater than 1% or low-quality bases greater than 40% were filtered; the final filtered data were called clean reads, after which the Q30 and GC contents were computed.

Alignment of clean reads was conducted based on the goat reference genome (GCF_001704415. 1_ARS1) using Bismark v0.23.0 [13] software. Statistical metrics, including bisulfite conversion and comparison rates, were also computed for each sample. The deduplicate_bismark program was then used to eliminate duplication reads, enabling the bismark_methylation_extractor and coverage2cytosine programs to isolate the unit point methylation information. The parameters were as follows:

bismark_methylation_extractor --samtools_path --bedGraph --CX --gzip --parallel --buffer_size

coverage2cytosine --split_by_chromosome --genome_folder -CX --gzip

The DNAm level was calculated as follows [14,15]:

$${Rm}_{average}=\frac{{Nm}_{all}}{{Nm}_{all}+{Nnm}_{all}}*100\%$$

where ${Nm}_{all}$ represents the number of methylated cytosine residues covering the site, and ${Nnm}_{all}$ represents the number of nonmethylated reads.

This study used the R package DMRcaller [16] to calculate and analyze the DMRs. For different contexts, different minProportionDifference parameters were used. The default CG was 0.2, the CHG was 0.1, and the CHH was 0.05.

### 2.5. DMR-Associated Genes (DMGs) and Functional Enrichment Analysis

BEDTools [17] was employed to determine DMR-related genes or other genome elements according to DMR genome position. In this study, genes or gene elements that overlapped with DMRs by at least 1 bp were considered DMR-associated genes or DMR-related elements. Furthermore, the DMG was subjected to GO enrichment analysis and KEGG functional annotation using DAVID “https://david.ncifcrf.gov/ (accessed on 29 September 2023)” and was considered to be significantly enriched at *p* < 0.05.

### 2.6. RT-qPCR Analysis

cDNA synthesis was conducted with the PrimeScript RT Reagent Kit with gDNA Eraser (Perfect Real Time) (Takara, Dalian, China). The following cDNA was obtained by reverse transcription: 2 µL 5× gDNA Eraser Buffer, 1 µL gDNA Eraser, 1 µg total RNA and RNase free dH_2_O up to 10 µL. The mixture was subsequently placed in a PCR machine for the reaction, which was performed at 42 °C for 2 min. Afterward, the reaction mixture was removed, and 4 µL RT Primer Mix, 4 µL 5× PrimeScript Buffer 2, 1 µL PrimeScript RT Enzyme Mix 1, and 1 µL RNase free dH_2_O were added. The mixture was subsequently placed in the PCR instrument again for the reaction, after which the reaction protocol was 37 °C for 15 min, 85 °C for 5 s, and 4 °C, the cDNA was then diluted to 10 ng/µL and stored at −20 °C.

Primers pairs were designed with Primer Premier 5.0 to validate the expression levels of key genes by real-time quantitative PCR (RT-qPCR) using GAPDH as an internal reference gene, All primers were diluted to 10 µM. RT-qPCR was carried out on a Roche LightCycler^®^480 II with 10 µL TB Green Premix Ex Taq II (Tli RNaseH Plus) (2× Conc), 0.8 µL upstream and downstream primers each, 2 µL cDNA template and 6.4 µL RNase-free dH_2_O. The reaction procedure was as follows: 95 °C for 3 min, followed by 40 cycles of 95 °C for 5 s, 60 °C for 20 s, and extension at 72 °C for 5 s and 40 cycles. Three replicates were determined for each sample, and the change in target gene expression was determined via the 2^−ΔΔCt^ method. The primer sequences are summarized in Table 1.

### 2.7. Bisulfite Sequencing PCR (BSP)

BSP was conducted to assess the DNAm level of the DMR region of key candidate genes. The genomic DNA was modified with sodium bisulfite using an EZ DNA Methylation-Gold^TM^ Kit (Cat.#D5005; Zymo Research, Los Angeles, CA, USA) with a uniform dilution of 200 ng/µL and stored at −20 °C. We used MethPrimer 2.0 “http://www.urogene.org/methprimer/ (accessed on 29 October 2023)” online analysis software to design BSP primers for the DMR region, which were then delivered to Sangon Biotech (Shanghai, China) for synthesis. The DMR region was further amplified by PCR using bisulfite-converted DNA as a template in 12.5 µL Zymo Taq^TM^ PreMix (Cat. #E2003; Zymo Research, Los Angeles, CA, USA), 1 µL upstream and downstream primers each, 2 µL gDNA, and 8.5 µL RNase-free dH_2_O. The reaction procedure was as follows: 95 °C for 10 min, followed by 40 cycles of 95 °C for 30 s, 52 °C for 30 s, 72 °C for 30 s, and 72 °C for 10 min. The PCR product was evaluated via 2% gel electrophoresis, and the qualified PCR products were placed in a PCR product purification kit (Cat.#BSC02M1A. Bioer Technology, Hangzhou, China) for purification, followed by ligation and cloning, and inserted into the pMD19-T vector (Cat. #6013; Takara, Osaka, Japan). Subsequently, the ligated product was transferred into DH5α receptor cells (Cat. G6016, AngYuBio, Shanghai, China), further coated on LA solid medium and cultured at 37 °C for 16 h. Afterwards, three white colonies were randomly picked from the medium, inoculated in LB liquid medium, and cultured for 3 h at 220 rpm/h. Afterward, PCR amplification was carried out in 10 µL 2× Taq PCR StarMix (Dye) (Cat# A012, GenStar, Beijing, China), 0.8 µL upstream and downstream primers each, 2 µL bacterial broth, and 6.4 µL RNase-free dH_2_O, with the conditions of the reaction described above. The PCR product was tested again via 2% gel electrophoresis, and the samples that passed quality control were then subsequently delivered to Sangon Biotech (Shanghai, China) for sequencing. The primer sequences are summarized in Table 2.

### 2.8. Statistical Analysis

The experimental data were preprocessed using Microsoft Excel, and independent sample *t* tests were conducted with SPSS 25.0. All values are shown as the mean ± SE, with a *p* value of 0.05 as the threshold.

## 3. Results

### 3.1. Genome-Wide Methylation Mapping

**Figure 1:**
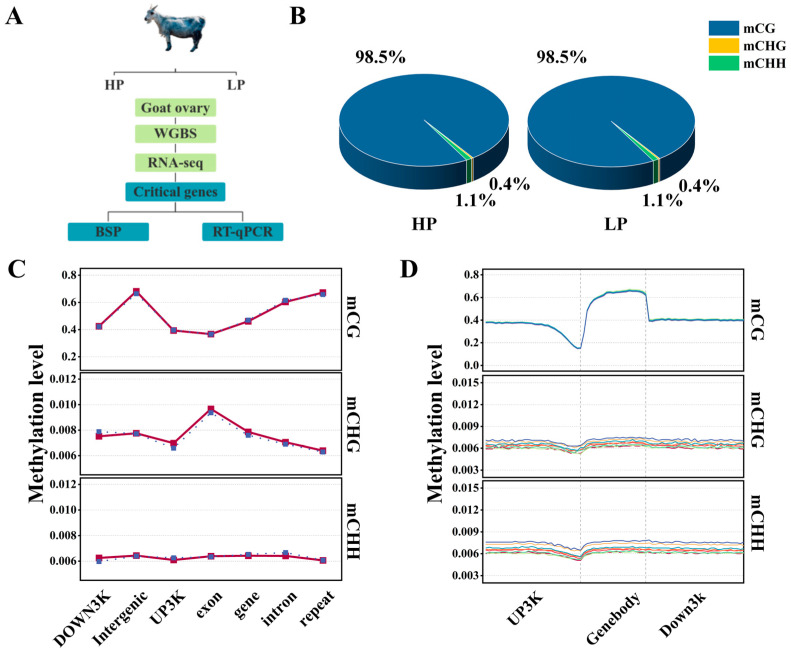
Genome-wide DNAm profiles of HP and LP Jining Grey goat ovaries. (**A**) Experimental design. (**B**) Mean proportion of diverse methylation contexts in the ovaries of Jining Grey goats. Blue, yellow and green represent methylated mCG, mCHH and mCHG, respectively. (**C**) Methylation levels in different genomic elements. Horizontal coordinates represent genomic elements, and vertical coordinates represent methylation levels; the value is the average methylation level within a gene element, with different colors representing different groups. (**D**) Distribution of DNAm level at 3K upstream/downstream. Horizontal coordinates show diverse regions, vertical coordinates represent methylation levels, with different colors representing different samples. Genebody: the active coding region of a gene from the beginning to the end of active coding (includes the exon and intron of the gene); UP3K: refers to 3000 bp before starting from the transcription start site (TSS); DOWN3k:refers to the transcription termination site (TTS) after 3000 BP.

In this study, the ovaries of HP and LP Jining Grey goats were used as tissue test samples, and whole-genome DNAm profiles of the ovaries of HP and LP Jining Grey goats were constructed via WGBS sequencing and RNA-seq data to screen key candidate genes. Finally, the methylation and expression levels were verified via BSP and RT-qPCR (Figure 1A). After the sequencing data were filtered, an average of approximately 1.16 billion clean reads were produced for each sample. The conversion rate after bisulfite treatment was >99%, the Q30 of the clean reads ranged between 90.16% and 91.94%, and the Unique mapping ratio accounted for 74.17~79.17% of the total reads. The percentage of Total_mC to all C sites was 2.98~3.66%. The sequencing data’s quality is depicted in Table 3. Bismark statistics of methylation sites showed that the genome-wide mC levels of 98.539 ± 0.010% for CG, 0.362 ± 0.002% for CHG, and 1.099 ± 0.009% for CHH in HP. In LP, the levels were 98.511 ± 0.035% for CG, 0.368 ± 0.009% for CHG, and 1.121 ± 0.028% for CHH. There was no obvious difference between the groups (Figure 1B), and there were significantly more methylated cytosine sites in CG than in CHG and CHH (where H is T, C or A). The DNAm levels of diverse genomic elements in the HP and LP groups of Jining Gray goats showed that there were diverse DNAm levels in the three contexts. In the CG, the intergenic exhibited the highest DNAm level, and the exon had the lowest methylation level. However, there was almost no methylation in the CHH or CHG contexts (Figure 1C). The gene body region exhibited high methylation, followed by the UP3K and Down3K regions, and the lowest methylation level was found near the transcription start site (TSS) locus (Figure 1D).

### 3.2. Differential Methylation Region (DMR) Identification

**Figure 2:**
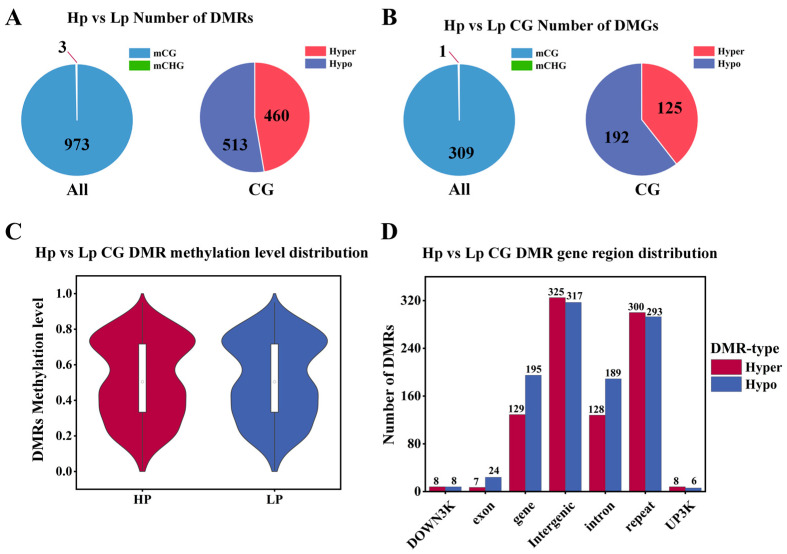
Identification of DMRs and DMGs. (**A**) Number of DMRs distributed in mCG, and mCHG, and number of hypo and hyper DMRs in CG. (**B**) Number of DMG distributions in mCG, and mCHG, and number of hypo and hyper DMG in CG. (**C**) Distribution of DMR methylation levels in CG. The horizontal axis indicates HP and LP groups, and the vertical coordinate indicates methylation level values. (**D**) DMR anchor region in CG. On the x-axis, each region’s type is indicated, while the number of hyper/hypo DMRs in each region is represented on the y-axis.

In this study, a total of 973 CG DMRs (hyper: 460; hypo: 513) and 3 CHG DMRs (hypo: 3) were identified between the HP and LP groups of the Jining Grey goats, and no DMRs were identified in the CHH population (Figure 2A). The identified DMRs were compared to the goat reference genome (GCF_001704415.1_ARS1), and a total of 310 differentially methylated genes (DMGs) were identified, 309 of which were found in the CG (hyper:125; hypo:192), while only one differentially methylated gene was found in the CHG (Figure 2B). The majority of DMGs belonged to the CG type (>99%), and this study evaluated CG methylation for the next analysis. The distribution of DNAm levels in DMRs showed that the mean DNAm levels ranged from 0.51 to0.53, with no significant differences between groups (Figure 2C). The DMR gene region showed that DMRs were mostly distributed in the intergenic region, with only a small number of DMRs distributed in the DOWN3K, exon, and UP3K regions (Figure 2D).

### 3.3. DMG Functional Enrichment Analysis

**Figure 3:**
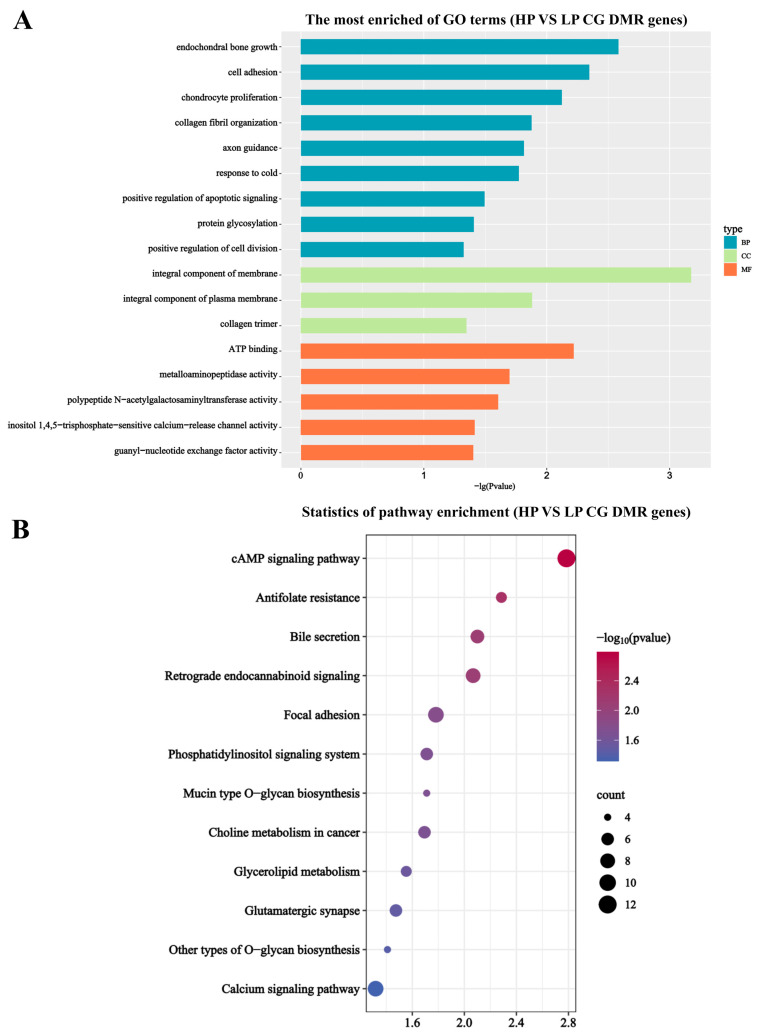
DMG functional enrichment analysis. (**A**) GO. horizontal coordinate represents −lg (*p* value), and vertical coordinate represents the corresponding GO terms. (**B**) KEGG: vertical and horizontal coordinate indicate enriched pathway and the corresponding pathway, respectively. The size of the dots indicates the amount of DMG contained in each pathway, and the color of the dots reflects the −log10 (*p*-value) of each pathway.

To determine the regulatory mechanism of genes related to litter size in Jining Grey goats, we conducted GO and KEGG functional enrichment analyses of 309 CG-type DMGs detected in the ovarian tissues of HP and LP Jining Grey goats in the present study. GO analysis showed that the DMGs were enriched mainly in biological processes, including endochondral bone growth, cell adhesion, chondrocyte proliferation, and collagen fibrils. In addition, the GO term “cellular component” was enriched mainly in the integral component of the membrane, integral component of the plasma membrane, and collagen trimer, whereas the GO term “molecular function” was enriched mainly in the GO term “molecular function” and was predominantly enriched in ATP binding, metalloaminopeptidase activity, and polypeptide N-acetylgalactosaminyltransferase activity (Figure 3A). KEGG analysis demonstrated that the DMGs were predominantly enriched in the ABC transporter, cAMP signaling pathway, antifolate resistance, and bile secretion pathway (Figure 3B).

### 3.4. Screening for DMG Associated with Litter Size Traits

**Figure 4:**
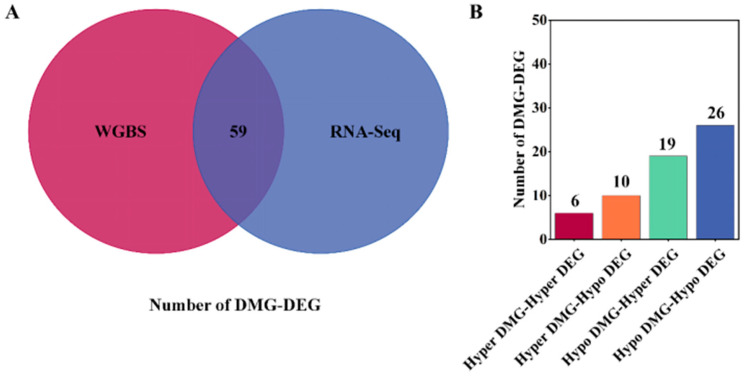
Combined DNAm and transcriptome analysis of HP and LP Jining Grey goat ovaries. (**A**) Venn diagram represents the intersection of DMG and DEG in WGBS and RNA-Seq; red represents DMG; and blue represents DEG. (**B**) DMG-DEG divided into groups according to hyper and hypo; vertical coordinate indicates the number of DMG-DEG in each group.

This study combined WGBS and RNA-Seq data to identify 59 differentially methylated and expressed genes (Figure 4A). Among these genes, 32 DMG methylation levels were positively correlated with expression levels and 29 DMGs were negatively correlated with expression levels (Figure 4B); the details are shown in Table 4. Finally, we identified eight key genes that might be related to litter size traits in Jining Grey goats through previous studies, and these have been previously reported in other studies. Eight key candidate genes were associated with *SERPINB2*, *NDRG4*, *CFAP43*, *LRP1B*, *EPHA6*, *TTC29*, *PDE11A*, and *PGF*. One DMR was identified in each of the *SERPINB2* and *NDRG4* genes located in the UP3K region, one DMR was identified in the HP group compared to the LP group, one DMR was identified in the *CFAP43* region located in the intro, and one DMR was upregulated. Another DMR was identified in the *LRP1B*, *EPHA6*, *TTC29*, and *PDE11A* regions located in the intro, and the methylation level was downregulated. Two DMRs were identified in the *PGF* region located in the exon and intro; and the methylation level was downregulated (Table 5).

### 3.5. Key Candidate Gene Validation

**Figure 5:**
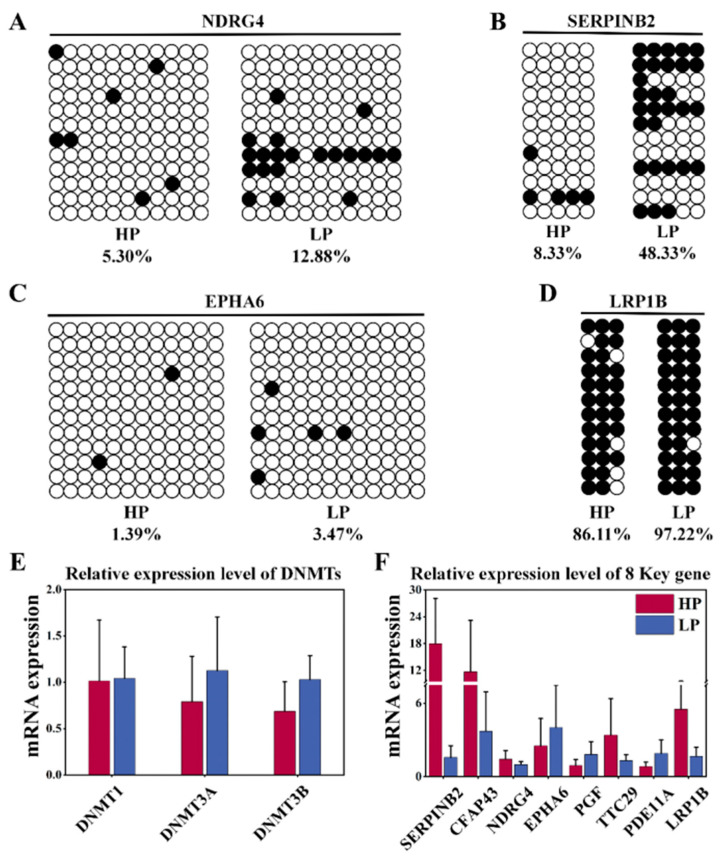
Key candidate gene validation. Methylation levels of (**A**) *NDRG4*, (**B**) *SERPINB2*, (**C**) *EPHA6*, and (**D**) *LRP1B* genes were validated by bisulfite sequencing PCR (BSP). Each circle represents a CpG dinucleotide, and black and white denote methylated and unmethylated sites, respectively. RT-qPCR was utilized to examine the mRNA expression of the (**E**) *DNMTs* in the ovaries and the (**F**) eight relative mRNA expressions of key candidate genes; mean ± SEM of 3 biological replicates of 2^−ΔΔCt^ value.

The robustness of WGBS sequencing data was verified by BSP experiments, in which four DMR fragments were randomly selected from key candidate genes (*NDRG4*: chr18:27599410-27599622; *SERPINB2*: chr24:62258787-62258914; *LRP1B*: chr2:79444158-79444322; and *EPHA6*: chr1:39785850-39786019) and subjected to bisulfite sequencing PCR (BSP) (Figure 5A–D). The findings showed that the DNAm levels within the DMR in the HP subgroup were lower than those in the LP subgroup for *NDRG4*, *SERPINB2*, *LRP1B,* and *EPHA6*. In summary, the results of the BSP experiments were in good agreement with the WGBS sequencing results, and the sequencing results were robust. The mRNA levels of eight key candidate genes were validated through RT-qPCR, and those of *DNMTs* (*DNMT1*, *DNMT3A,* and *DNMT3B*) were detected. Notably, the expression levels of *DNMTs* (*DNMT1*, *DNMT3A,* and *DNMT3B*) in the HP group were lower compared to LP group (Figure 5E). The expression levels of the *SERPINB2* and *LRP1B* genes were upregulated, while the expression levels of the *PGF*, *EPHA6*, *NDRG4*, *CFAP43*, *TTC29* and *PDE11A* genes were downregulated (Figure 5F). In summary, *DNMT* expression was downregulated in the HP group, and the data from the BSP and RT-qPCR experiments were in good agreement with the sequencing results, meaning the findings are reliable.

## 4. Discussion

DNAm constitutes a prominent aspect of epigenetic regulation and plays a vital role in modulating gene expression [31]. Currently, genome-wide methylation profiles of pig [11,32], goat [9], and sheep [10] ovaries have been intensively studied. While genome-wide DNAm has been described in the ovaries of Jining Grey goats [9] and Saanen goats [12], there are still many unknowns about the epigenetic mechanisms of DNAm on litter size traits in goats, which have not been detected in studies on genome-wide DNAm and transcriptome-wide changes in litter size traits in goats. First, we assessed the genome-wide DNAm profile of the ovaries of Jining Grey goats using WGBS technology and found that methylated cytosine sites accounted for 2.98–3.66% of all cytosine sites and that the proportion of CG-type methylated sites in the genome was much greater than that of CHG and CHH, which is in agreement with the findings of other species [2,10,33,34,35,36]. It has been reported that the overall methylation level of the UP2K region in the ovary of Jining Grey goats is low and flat, the DNAm level increases after TSS, and the gene body region shows a high methylation level, which declines until after TTS [9]. This finding aligns closely with the results obtained in this study, in which the methylation level of cytosine sites near the TSS was significantly lower than that of the UP3K and DOWN3K regions in the ovaries of the Jining Grey goat, whereas the gene body region likewise showed a high degree of methylation, a result that is consistent with the DNAm patterns previously observed in the ovaries of Hu sheep [10] and pig [36] ovaries. A total of 976 DMRs and 310 DMGs were identified, and no obvious difference was observed in the DNAm level of DMRs between the two groups; most of the DMRs were detected within the intergenic region, and a small percentage of them were distributed in the DOWN3K, exon, and UP3K regions, a result that is similar to that of previous studies on the ovaries of Hu sheep [10] and pigs [36]. Pathway analysis revealed that DMGs were involved in cell division and proliferation through the p38MAPK and cAMP signaling pathways. No relationship was observed between tissue functions and specific pathways in the ovary, which may be attributed to limited gene annotation in the goat genome.

DNA methyltransferase (*DNMT*) genes are key genes involved in the construction of mammalian DNAm patterns [37]. Previous studies have shown that *DNMT3a* may have wide-ranging effects on reproductive functions [38], and we found that the mRNA expression of *DNMTs* was lower in the HP group compared to the LP group; therefore, we hypothesized that *DNMTs* could mediate the methylation levels of genes related to litter size traits in Jining Grey goats. 

A total of eight key candidate genes that may be associated with litter size traits in Jining Grey goats were identified in this study (*SERPINB2*, *NDRG4*, *CFAP43*, *LRP1B*, *EPHA6*, *TTC29*, *PDE11A*, and *PGF*). Hypermethylation of promoters is usually associated with gene repression, and the DMRs of *SERPINB2* and *NDRG4* were found to be located within the promoter region in our research. *SERPINB2* is a serpin family B member. Prior reports have shown that in porcine ovarian granulosa cells after lipopolysaccharide (LPS) stimulation, *TNFα* plays an essential role in cell proliferation by activating the Erk1/2 pathway, which mediates the upregulation of *SERPINE1* and *SERPINB2* expression [22]. In the present study, DMR hypomethylation in the promoter region of *SERPINB2* promoted gene expression, which may have affected litter size traits in Jining Grey goats. The *NDRG4* gene has been reported to affect the "adhesive switch" by producing epigenetic silencing through promoter hypermethylation, and this gene could be a potential mechanistic biomarker for breast cancer [27]. The *NDRG4* gene, part of the N-myc downregulated gene family and belonging to the alpha/beta hydrolase superfamily, has garnered attention in recent studies for its potential role in estrogen-regulated embryo implantation in mice [26]. However, recent studies have shown that methylation of promoter regions can also activate gene expression [39]. Hypermethylation of the promoter region of the *FoxA2* gene promotes its expression and thus regulates the development of endoderm in cells [40]. Herein, similar results were found for the DNAm level and expression level of DMR in *NDRG4*. Moreover, GO functional analysis indicated that *NDRG4* was enriched in the vesicle docking term, and we further speculate that *NDRG4* might affect its litter size traits through vesicle docking. The effect of DNAm in the gene body region on the regulation of gene expression is more complicated [12,41]. The *CFAP43* gene, alternatively recognized as *WDR96*, exhibits widespread expression across gonadal tissues, and early studies have shown that mutations in the *CFAP43* gene cause infertility in Trypanosoma and humans [19]. Meanwhile, indel mutations in *CFAP43* in white cashmere goats from northern Shaanbei can affect their litter size [5] and body size traits [18]. A GWAS showed the *LRP1B* gene to be a key candidate gene for teat number in Luzhong meat sheep [20], following a study that showed a correlation between litter size and teat number [42]. The *EPHA6* gene is also known as ephrin type-A receptor 6, and previous studies have shown that *EPHA6* is a key candidate gene for prolificacy traits in Polish goats [7]. *TTC29* is involved in cilium movement and cilium organization. Chunyu Liu et al. [43] showed that biallelic mutations in *TTC29* can cause male infertility as well as asthenoteratospermia in humans and mice. The *PDE11A* gene encodes the PDE protein superfamily member and is associated with testicular germ cell tumors (TGCTs) [28,29]. Smoking can alter the overall methylation status of *PDE11A* in human spermatozoa and affect its transcription, which in turn affects male fertility [30]. Placental growth factor (*PGF*) enables growth factor activity and is closely related to fetal development. *PGF* has been identified as an important vascular growth factor in the placenta and contributes to placental and fetal development [24]. Apart from its roles in angiogenesis and modulating the growth and migration of various cell types, the presence of a CPG island hypermethylated on *PGF* exon 7 in the fetal growth restriction (FGR) placenta leads to the downregulation of *PGF* expression, which further regulates trophoblast cell proliferation and migration, affecting placental development and function [23]. Recent research has demonstrated that polymorphisms in the gene encoding placental growth factor (*PGF*) are strongly associated with stillbirths as well as calving ease in German dairy cows [25]. Herein, two DMRs were identified on the *PGF*, both of which showed hypomethylation and downregulated mRNA expression in the HP group, while GO analysis demonstrated that the *PGF* was enriched in the positive regulation of the cell division process. However, little has been reported on how DNAm of *CFAP43*, *LRP1B*, *EPHA6*, *TTC29*, *PDE11A* and *PGF* affects the litter size traits of female animals.

In this study, hypomethylation of the promoter region induced *SERPINB2* expression, while repressing *NDRG4* expression; hypermethylation within the gene body region repressed *CFAP43* expression; and hypomethylation within the gene body region promoted *LRP1B* expression and repressed *EPHA6*, *TTC29*, *PDE11A* and *PGF* expression. Therefore, in this study, we identified eight genes, *SERPINB2*, *NDRG4*, *CFAP43*, *LRP1B*, *EPHA6*, *TTC29*, *PDE11A*, and *PGF*, as key candidate genes regulating litter size traits in Jining Grey goats, and the DMRs on these genes may directly or indirectly regulate phenotypic differences between HP and LP Jining Grey goats. However, the epigenetic mechanism through which these genes regulate litter size traits in Jining Grey goats still needs further study.

## 5. Conclusions

In this work, genome-wide DNAm profiles of ovarian tissues from HPs and LP Jining Grey goats were constructed, and it was concluded that DNAm modification is a potential factor affecting Jining Grey goats. Further studies showed that nine methylation modification sites (SERPINB2: chr24_62258801_62259000, NDRG4: chr18_27599201_27599400, CFAP43: chr26_27046601_27046800, LRP1B: chr2_79720201_79720400, EPHA6: chr1_40088601_40088800, TTC29: chr17_59385801_59386000, PDE11A: chr2_117418601_117418800 and PGF: chr10_ 16913801_16914000; chr10_16916401_16916600) may regulate lambing traits in Jining Grey goats by influencing gene expression to further affect ovarian development. A better understanding of the influence of epigenetics on litter size traits is likely to aid increased animal prolificacy.

## Figures and Tables

**Table 1:** Primer information for RT-qPCR.

Primer Names	Sequences (5′-3′)		Product Length (bp)	Gene Bank
	Forward	Reverse		
**GAPDH**	CGGCACAGTCAAGGCAGAGAAC	CACGTACTCAGCACCAGCATCAC	115	XM_005680968.3
**DNMT1**	CCTGACTCCACCTACGAAGACC	CTACTTGCTCCACCACGAACTG	127	XM_018051019.1
**DNMT3A**	CCGCATTGTGTCTTGGTGGATG	AGAACTTGCCGTCTCCGAACC	86	XM_018055548.1
**DNMT3B**	TTGACTTGGTGATTGGTGGAAGC	CGAGTGTAATTCAGCAGGTGGTAG	124	XM_018057711.1
**PGF**	TGAGACTGTTCACTTGCTTCCTG	GCTGCGGCTCCACACTTG	131	XM_005686088.3
**EPHA6**	AGGATACAGTGGCTACAGTCAGAAG	ACGGCTGCGGTGGCTATC	105	XM_013972057.2
**NFASC**	ACGACAGCCTGGTGGACTATGG	TCCGTCTCCTCCTTGTCCTTCTTG	103	XM_018060022.1
**NDRG4**	CCAACCACCACGACCTTCCTG	AGCCTTCGGTCCTTCAAGTATGC	137	XM_018062073.1
**LRP1B**	GCACTGCGACTCTGATGATGACTG	ATCTGCCACTGGAACAACTGAACTG	98	XM_018062463.1
**LOC102181552**	TGTTCCTGCTGCTTCCAGATGG	GTACACCTCCACGTCATCCTCAC	134	XM_005697318.2
**CFAP43**	GCAAGGAAGCAGGAGGAGAGG	CCGCCAGTAAGTTGTCATAGTGTTC	100	XM_018041743.1
**TTC29**	GCATTAGCAGTCCTCAACACTTACG	CCGTGGCTTCATAGGCTCTCC	85	XM_005691202.2
**PDE11A**	CTCTATGGAACCTCTGCCACCTTG	ATGTTGTGACCCTCGCTCTGAAG	80	XM_005675916.3

**Table 2:** Primers sequences of DNA-methylation-related genes.

Region	Sequences (5′-3′)	Product Length (bp)	Associate Gene	Gene Bank
**chr18:27599410-27599622**	AAATATTTGTGTTTGGAGTTATTTT	213	*NDRG4*	NC_030825.1
	TACCCCAATCTTTATTTAAATTCCC			
**chr24:62258787_62258914**	TTTAGATTTAGTGATTAGATTAGTGGT	128	*SERPINB2*	NC_030831.1
	AACAATAAAACCTAACTCCATACC			
**chr2:79444158-79444322**	AAAATTTATGTAAATGTTAGAAGTTT	165	*LRP1B*	NC_030809.1
	AATAATTAAATAACATCACCAACTC			
**chr1:39785850-39786019**	TTATTTAGGTTAGGGGTTGAGGG	170	*EPHA6*	NC_030808.1
	ACACTAACAAAACCAAAAACAAAAC			

**Table 3:** Whole-genome DNA bisulfite sequencing data.

Groups	Sample	Clean Reads ^1^	Q30 ^1^ (%)	Total Mapping Reads ^1^ (%)	Unique Mapping Ratio ^1^ (%)	Bisulfite Conversion Rate ^1^ (%)	Total_mC ^1^ (%)
HP	HP-O1	1139474436	91.39	85.54	79.17	99.54	3.50
	HP-O2	1148095570	91.62	83.5	77.57	99.51	3.43
	HP-O3	1124419076	91.21	84.53	78.40	99.51	3.55
	HP-O4	1185419974	91.31	80.05	74.17	99.47	3.30
LP	LP-O1	1234628940	91.94	83.81	77.90	99.53	3.24
	LP-O2	1120935898	90.94	83.48	77.42	99.53	3.48
	LP-O3	1341873656	91.21	84.18	77.81	99.51	2.98
	LP-O4	1042249584	90.16	82.49	76.80	99.47	3.66

^1^ Clean base (Gb): total number of unambiguous base calls in the sequences; clean reads: total number of sequence reads after filtering for quality and contaminants; unique mapping ratio (%): proportion of clean reads that aligned uniquely to the reference genome out of the total clean sequences; bisulfite conversion rate (%): proportion of clean reads that underwent successful bisulfite conversion relative to the total amount of methylation observed in the clean sequences aligned to the reference genome; total mC (%): proportion of methylated cytosines within the clean sequences aligned to the reference genome relative to the total number of clean sequences aligned to the reference genome.

**Table 4:** Genes obtained from unite analysis of Whole-Genome Bisulfite Sequencing (WGBS) and RNA-Seq.

Group	Genes	Gene Counts
Hyper DMG-Hyper DEG	*NFASC* *,* *GLT1D1* *,* *DOCK10* *,* *JAKMIP3* *,* *GRM5* *,* *GRIK2*	6
Hyper DMG-Hypo DEG	*CR2, CDH20, **CFAP43*** [5,18,19], *DAGLA, GRM8, ZNF804B, RELN, TRHDE, LOC102172692, CLEC9A*	10
Hypo DMG-Hyper DEG	*ISM1, MMP16, NCAM1, USH2A, GALNT9, VAT1L, UPK1A, **LRP1B*** ^1^ [20,21], *FMNL2, CTNND2, FSD2, MTCL1, **SERPINB2*** ^1^ [22], *ADAM12, DLG2, LOC102170378, GSTM3, GRIK2, TMEM176B*	19
Hypo DMG-Hypo DEG	*CADM2, **EPHA6*** ^1^ [7], *C1H3orf52, **PGF*** ^1^ [23,24,25], *ALDH1A2, LOC108637248, COL14A1, **TTC29*** ^1^ [3], ***NDRG4*** ^1^ [26,27], *HPN, ARHGAP27, **PDE11A*** ^1^ [28,29,30], *MFSD6, MYO7B, JAG2, CDH20, TRPM8, C3H1orf87, C3H1orf226, COL28A1, SUN3, CRACR2A, UNC5A, RFX2, FBXO16, LOC108634594*	26

^1^ Genes in bold are selected key candidate genes, and relevant references are included in [].

**Table 5:** DMR methylation levels of 8 critical genes of HP and LP Jining Grey goat.

Gene	Chr ^1^	Start	End	Element	Meth-Direction	Meth Diff ^1^	Q-Value
*SERPINB2*	24	62258801	62259000	UP3K	Hypo	−0.331	1.09 × 10^−3^
*NDRG4*	18	27599201	27599400	UP3K	Hypo	−0.326	4.35 × 10^−5^
*CFAP43*	26	27046601	27046800	intro	Hyper	0.435	1.65 × 10^−2^
*LRP1B*	2	79720201	79720400	intro	Hypo	−0.312	2.81 × 10^−2^
*EPHA6*	1	40088601	40088800	intro	Hypo	−0.309	1.19 × 10^−4^
*TTC29*	17	59385801	59386000	intro	Hypo	−0.328	1.36 × 10^−5^
*PDE11A*	2	117418601	117418800	intro	Hypo	−0.403	3.33 × 10^−5^
*PGF*	10	16913801	16914000	exon	Hypo	−0.309	8.42 × 10^−4^

^1^ Chr: chromosome, Meth diff: difference in DNAm levels between HP and LP.

## Data Availability

All WGBS and RNA-seq data generated in this study were submitted to the NCBI SRA database under BioProject: NO.PRJNA1068426 (WGBS) and NO.PRJNA1068677 (RNA-seq).
